# Geochemical characterization data of harbors dredged sediments in the Occitanie region (southern France)

**DOI:** 10.1016/j.dib.2024.110509

**Published:** 2024-05-11

**Authors:** Garry Dorleon, Isabelle Techer, Sylvain Rigaud

**Affiliations:** UPR 7352 CHROME, Site Hoche - Université de Nîmes, Laboratoire Géosciences de l'Environnement, 1 place du président Doumergue, 30000 Nîmes, France

**Keywords:** Waste management, Circular economy, Coastal sediment quality, Physicochemical properties, Dredging, harbors sediments

## Abstract

Over the last 15 years, numerous analyses of sediment from the Mediterranean harbors of Occitanie (Southern France) has been made before dredging operations in order to assess geochemical quality of dredged sediment and define the extend of dredging project and the potential fate of dredged sediment (sea dumping vs management on land). However, these data are today scattered, printed and stored as archives, and not directly accessible. With time, those data are expected to be lost for the community whereas they constitute an irreplaceable and mobilizable knowledge base to address the challenges of the circular economy. Characterization data aid in developing regulations for better land-based management of dredged sediments. Existing data are also needed to define pollutant limits in sediments for different uses of marine resources. The collection of these data can thus offer a unique opportunity to assess the geochemical quality of dredged marine sediments and their determining factors. The dataset collected is composed of geochemical characteristics of 146 marine sediments wastes collected before dredging operations between 2010 and 2021. The sampling was designed to capture the large diversity of sediment distribution in harbors of southern France. The dataset contains a wide range of variability in the composition characteristics of dredged sediment (dry matter, organic matter, total nitrogen and phosphorus, sulphate, chloride, trace metals and organics elements). Because the dataset provides information about the characteristics defining the geochemical quality of dredged sediments, it can be used further for research, waste management or dredged sediment valorization, and represent a great interest to other researchers, harbors managers and stakeholders in search of references on the geochemical quality of dredged sediments for their reuse.

Specifications Table

**Table:** 

Subject	Chemistry and Environmental Sciences
Specific subject area	Geochemical composition of dredged marine sediment
Type of data	Table, Figure
Data format	Raw, Analyzed
Data collection	Raw data were acquired, from 10 harbors in Occitanie (southern France), between 2010 and 2021, as the result of measurements, sampling and analysis before sediments dredging operations. Analysed data included compositional parameters such as hydrogen potential, particle size, dry matter and organic matter contents, total phosphorus and nitrogen, leached chlorides, sulphates and fluorides, metalloids and metals contents and organics contaminants concentrations.
Data source location	Institution: Université de Nîmes City/Town/Region: Occitanie (Southern France) Country: France The dataset provides GPS coordinates of the sampling points.
Data accessibility	Repository name: Recherche Data Gouv Data identification number: 10.57745/CLDJVQ Direct URL to data: https://doi.org/10.57745/CLDJVQ Licenses of use: etalab 2.0 (https://spdx.org/licenses/etalab-2.0.html)

## 1 Value of the Data

• The dataset provides information about the physicochemical composition diversity of dredged marine sediments. these data are useful for developing regulatory framework to improve circular management of dredged sediments. These data are also needed to define pollutant limits in sediments for different uses of marine resources.
• The dataset in this article provides information about the characteristics defining the geochemical quality of dredged sediments studied. It can be used further for research, improve waste management, and/or dredged sediment valorization.
• The dataset contains a wide range of variability on the composition of marine sediments, can be merged with other databases and represent a great interest to other researchers, harbors managers and stakeholders in search of references on the quality of dredged sediments for their reuse in various applications ranging from construction to plant growth medium.

## 2 Background

In this vibrant narrative of climate action, recycling emerges as a beacon of hope and ingenuity. A myriad of initiatives, driven by a shared commitment to environmental stewardship, has already left indelible marks on conservation efforts. Countless works have been dedicated to recycling, becoming the catalysts for transformative change.

In the Occitanie region (southern France), anthropogenic activities and the sedimentary dynamics in and around harbors, provides the movement of significant quantities of solid materials and siltation phenomena [1]. In order to maintain and develop harbors activities and sustain the economy of littoral territories, frequent maintenance dredging operations emerged in recent years, and considerable volume of sediments are dredged.

French regulations come to govern the management of dredged marine sediments, considering them ‘safe’ and possibly to be dumped at sea or ‘contaminated’ and needed to be treated on land as waste [2]. However, sediments can be considered as valuable resources but they remain today lowly reused. This non-reuse can particularly be explained by the lack of available data on the composition of dredged sediments or the inaccessibility to these data when they are available.

Before any intervention (dredging, valorization or disposal), the knowledge and the characterization of the geochemical composition of the sediment are necessary. The objective in this paper was to provide dredged marine sediment samples [3] that represented the diversity of the wide range of geochemical composition of these materials most commonly considered as waste and their potential for recovery in a circular economy perspective.

## 3 Data Description

**Fig. 1:**
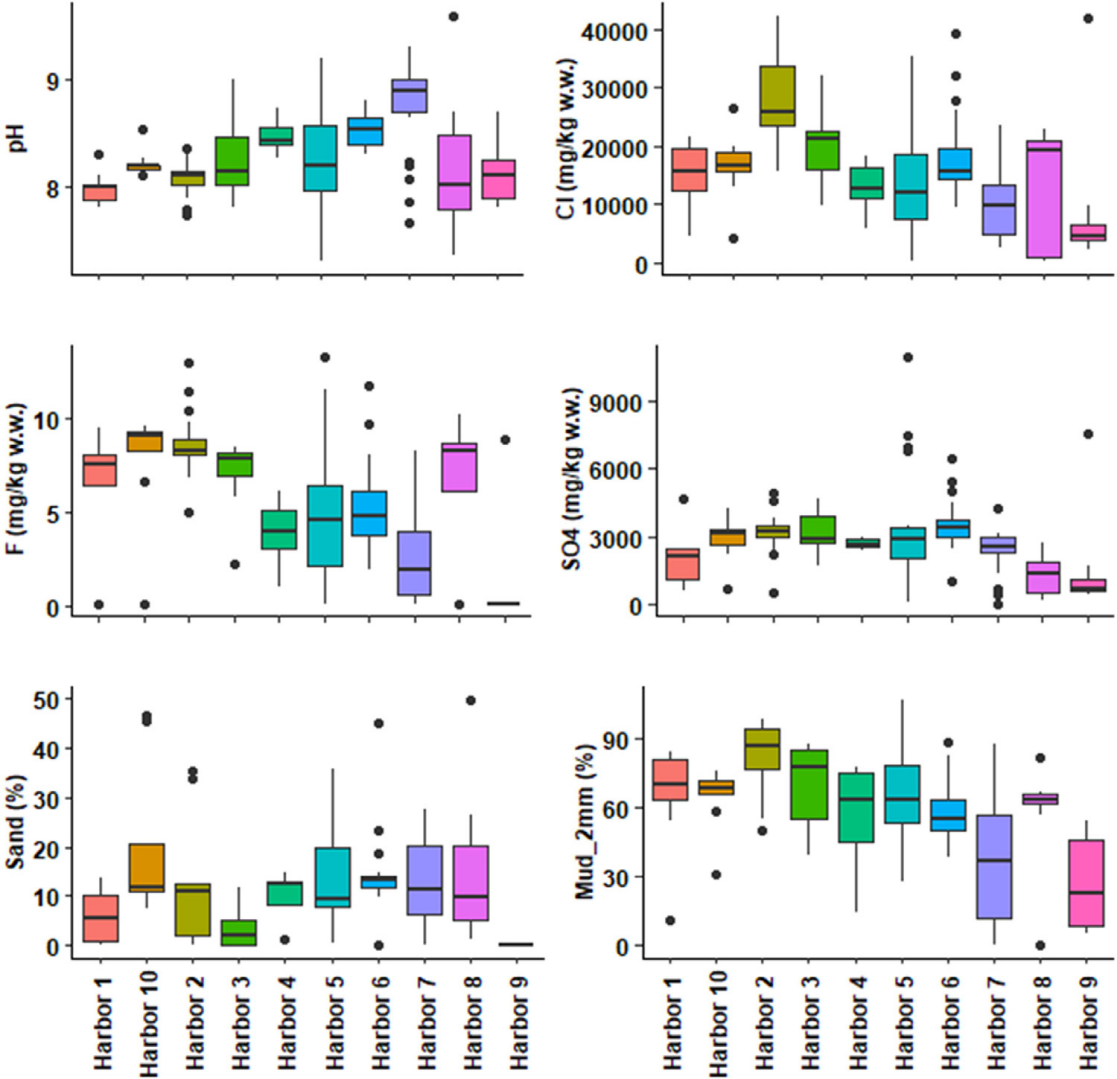
Boxplots of hydrogen potential (pH), chloride (Cl), fluoride (F), sulphate (SO4), Mud_2 mm (vase content on fraction < 63 µm) and sand content by type of sediment (harbor). (Whiskers represent 1.5 times the interquartile range) (w.w.: wet weight).

**Fig. 2:**
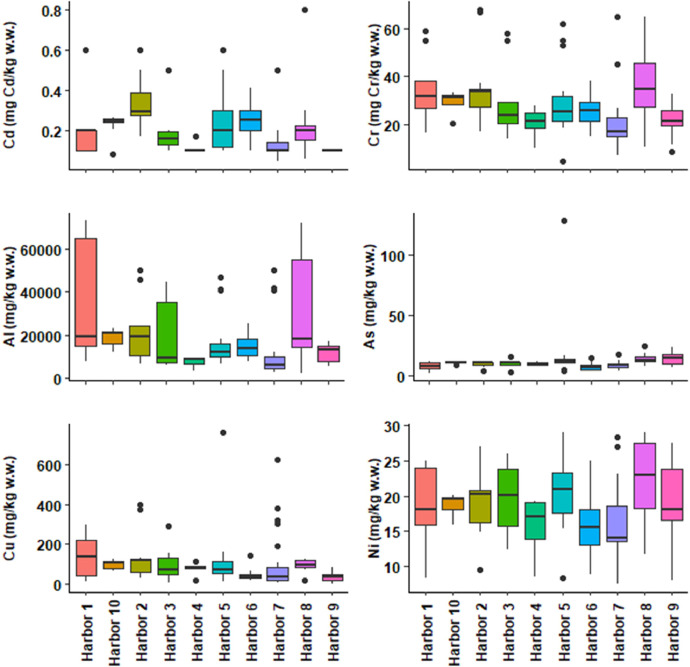
Boxplots of cadmium (Cd), aluminum (Al), chromium (Cr), arsenic (As), copper (Cu) and nickel (Ni) content by type of sediment (harbor). (Whiskers represent 1.5 times the interquartile range) (w.w.: wet weight).

**Fig. 3:**
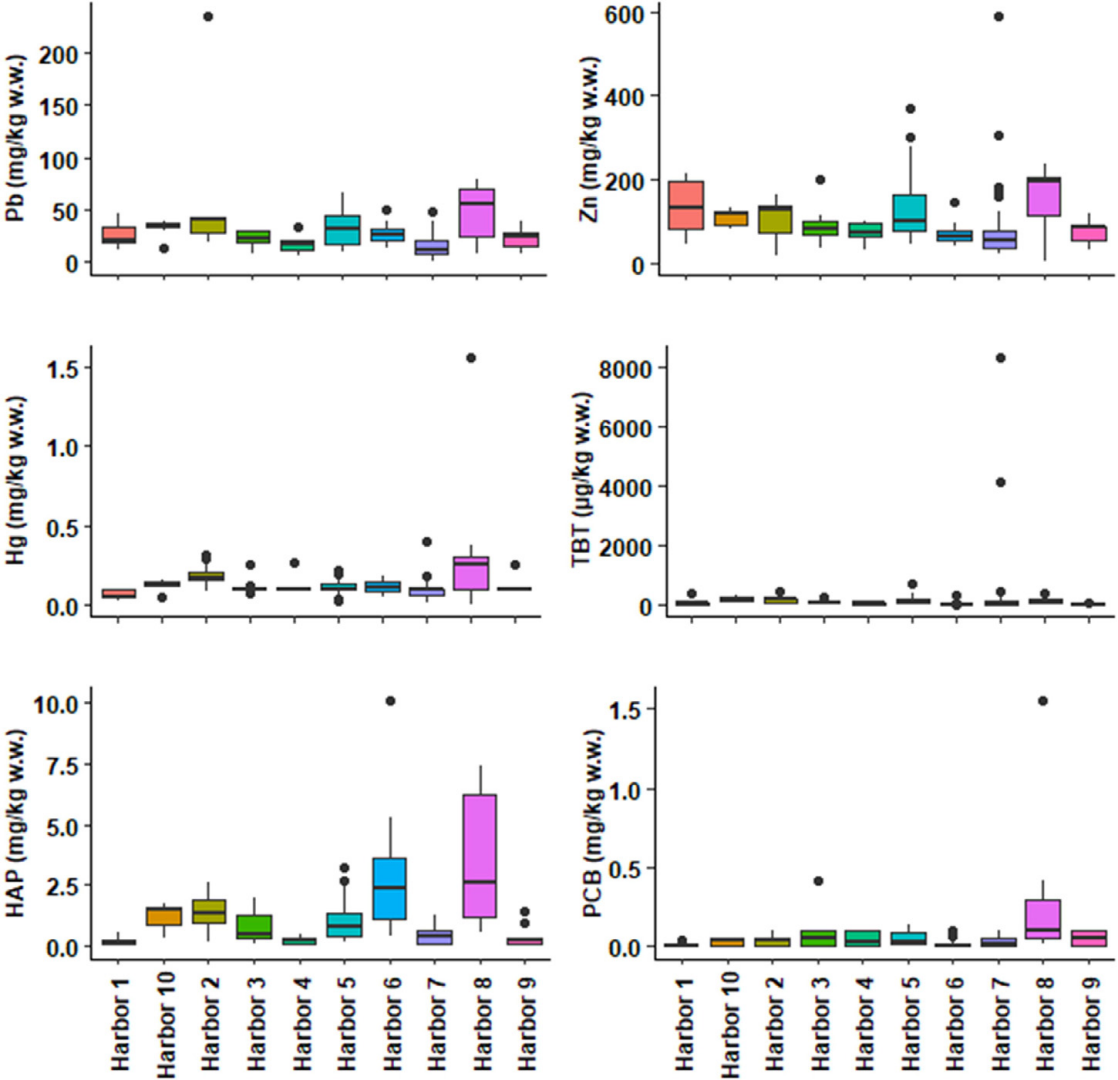
Boxplots of lead (Pb), zinc (Zn), mercury (Hg), tributyltin (TBT), polycyclic aromatic hydrocarbons (HAP) and polychlorinated biphenyls (PCB) content by type of sediment (harbor). (Whiskers represent 1.5 times the interquartile range) (w.w.: wet weight).

**Fig. 4:**
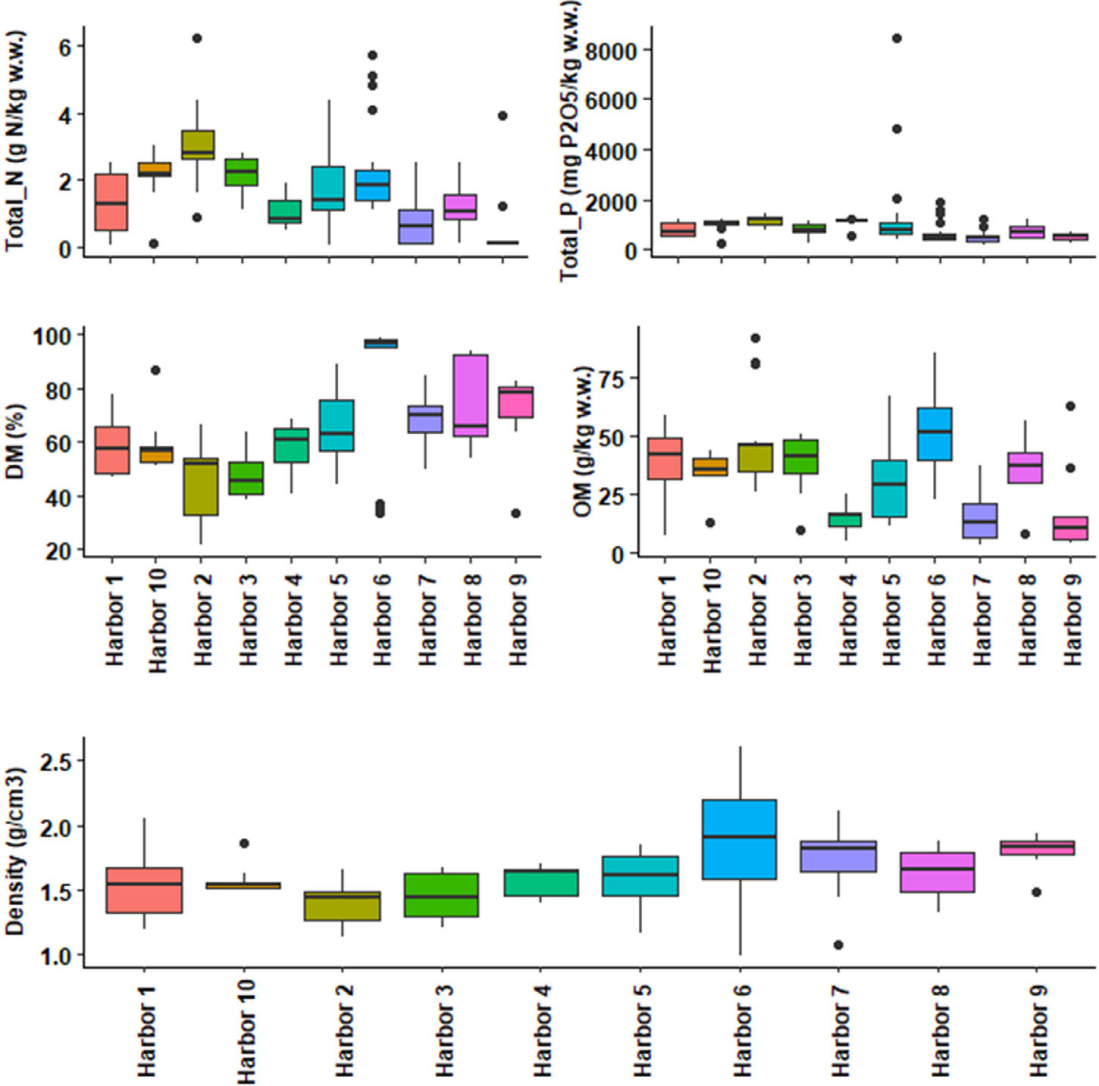
Boxplots of total nitrogen (Total_N), phosphorus total (Total_P), dry matter (DM), organic matter (OM) content and bulk density (Density) by type of sediment (harbor). (Whiskers represent 1.5 times the interquartile range) (w.w.: wet weight).

**Fig. 5:**
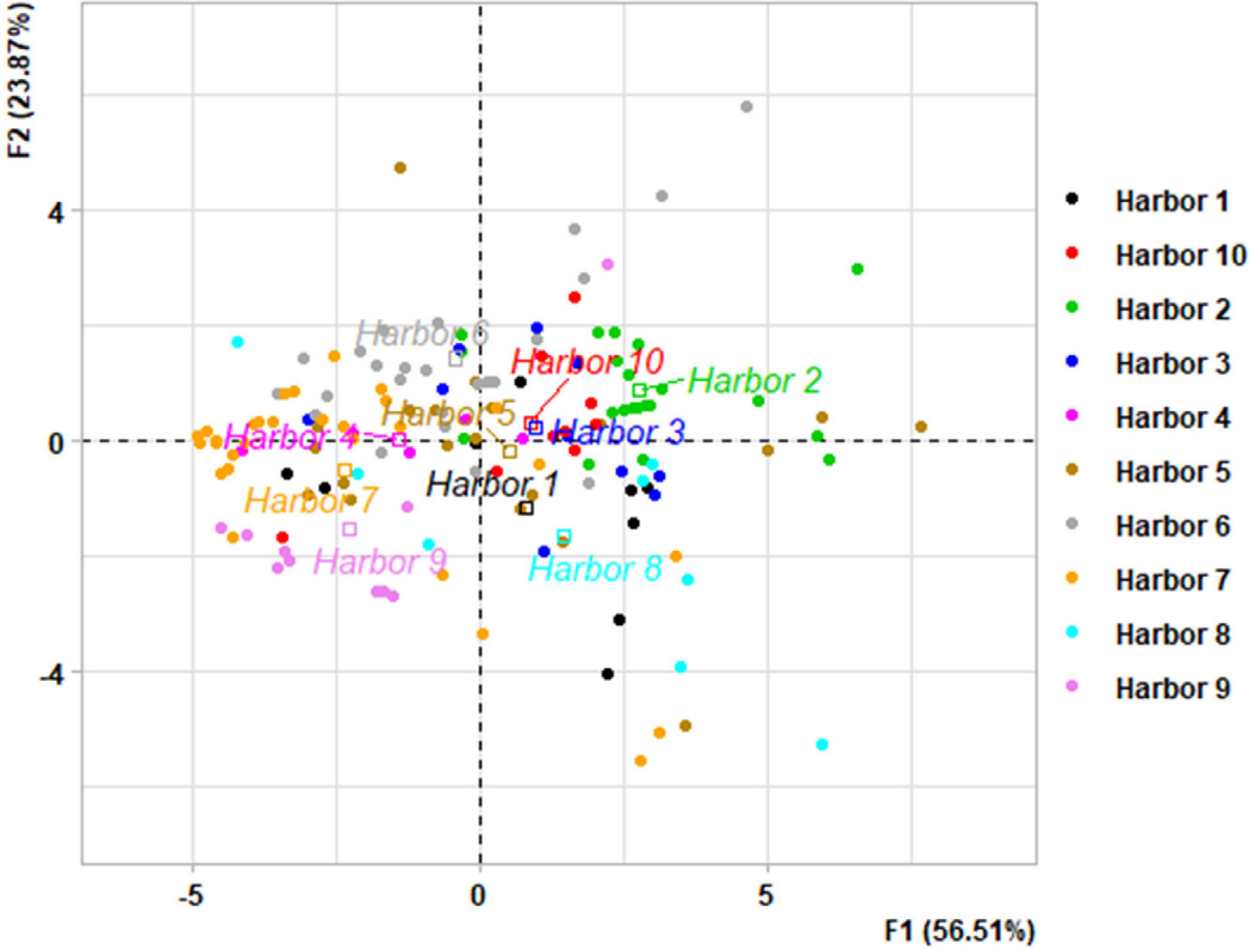
Projection of the 146 dredged marine sediment on the plane defined by the first two components of a principal component analysis of the composition parameters. Samples colored by type of sediment (harbor).

**Fig. 6:**
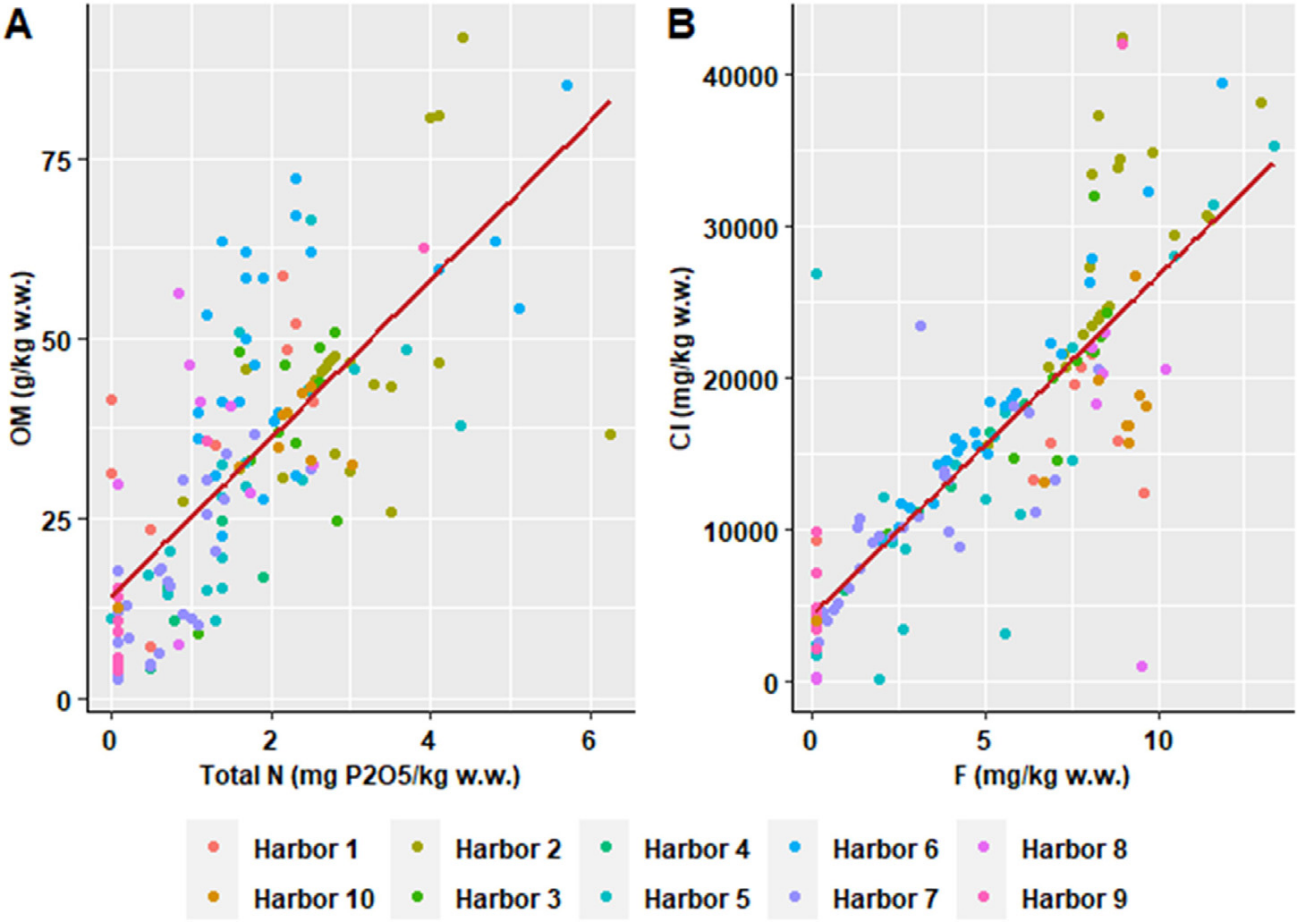
Relation between (A) organic matter and total nitrogen content and (B) chloride and fluoride of dredged marine sediments (red lines indicate linear regressions) (w.w.: wet weight).

This dataset contains tables and figures that describe 23 geochemical composition parameters of 146 marine sediments wastes, sampled between 2010 and 2021 in 10 harbors in the Occitanie region, southern France. Table 1 includes mean values of geochemical composition parameters by type of sediments or harbors. Table 2 shows a correlation matrix between each pair of composition parameters. boxplots of all geochemical composition parameters, representing the variability in composition by type of sediment studied are also presented. Fig. 1 shows boxplots of hydrogen potential (pH), chloride (Cl), fluoride (F), sulphate (SO_4_), vase content on fraction < 63 µm (Mud_2 mm) and sand content by type of sediment (harbor); Fig. 2 shows boxplots of cadmium (Cd), aluminum (Al), chromium (Cr), arsenic (As), copper (Cu) and nickel (Ni) content; Fig. 3 shows boxplots of lead (Pb), zinc (Zn), mercury (Hg), tributyltin (TBT), polycyclic aromatic hydrocarbons (HAP) and polychlorinated biphenyls (PCB) content; Fig. 4 shows boxplots of total nitrogen (Total_N), phosphorus total (Total_P), dry matter (DM), organic matter (OM) content and bulk density (Density) by type of sediment (harbor). A projection of the dredged marine sediments on the plane defined by the first two components of a principal component analysis of the geochemical composition parameters and the relations between some parameters such as organic matter and total nitrogen, chloride and fluoride contents are respectively illustrated in Fig. 5, Fig. 6. The dataset includes the location of the sampling points, sediments type and the geochemical analysis results of the 146 dredged marine sediments sampled. The raw data for parameters collected are available in the repository DOI: 10.57745/CLDJVQ.

**Table 1:** Mean values of geochemical composition parameters for each type of dredged marine sediment (pH: hydrogen potential; Cl: chloride; F: fluoride; SO4: sulphate; Cd: cadmium; Al: aluminum; Cr: chromium; As: arsenic; Cu: copper; Ni: nickel; Pb: lead; Zn: zinc; Hg: mercury; TBT: tributyltin; HAP: polycyclic aromatic hydrocarbons; PCB: polychlorinated biphenyls; Sand: sand content; Mud_2mm: silt content on fraction < 63 µm; Density: bulk density; Total N : total nitrogen; Total P: phosphorus total; DM: dry matter content; OM: organic matter content).

Sediment	Sample size	pH	Cl	F	SO4	Cd	Cr	Al	As	Cu	Ni	Pb	Zn	Hg	TBT	HAP	PCB	Sand	Mud 2mm	Density	Total N	Total P	DM	OM
Harbor 1	9	7,99	14,761,01	6,14	2011,41	0,20	35,13	32,960,78	7,71	145,57	18,44	25,87	136,18	0,07	54,99	0,23	0,01	5,93	65,14	1,55	1,28	767,03	59,38	37,68
Harbor 2	22	8,08	28,115,53	8,71	3213,52	0,32	33,69	20,718,21	9,70	121,34	19,06	44,37	106,39	0,18	146,34	1,43	0,04	9,79	82,95	1,39	3,06	1122,29	45,57	46,44
Harbor 3	10	8,25	20,276,47	7,09	3142,36	0,19	29,46	19,071,10	10,06	98,35	19,78	22,39	91,44	0,11	53,87	0,79	0,08	3,64	70,11	1,45	2,19	800,32	46,94	37,70
Harbor 4	5	8,48	12,910,44	3,86	2675,00	0,11	20,58	7624,00	9,98	77,46	15,56	17,97	71,30	0,13	44,10	0,28	0,05	9,87	55,06	1,58	1,06	1039,40	57,62	14,31
Harbor 5	20	8,29	13,970,79	4,80	3427,98	0,24	28,91	16,557,71	17,37	110,89	20,29	33,19	136,49	0,12	139,79	1,04	0,05	13,44	64,20	1,58	1,71	1408,08	65,53	29,16
Harbor 6	24	8,54	18,162,16	5,28	3470,92	0,26	25,54	14,395,54	7,08	43,49	15,45	26,75	66,14	0,11	13,92	2,95	0,02	14,25	57,72	1,87	2,27	657,70	84,10	50,17
Harbor 7	29	8,72	9811,59	2,62	2339,65	0,12	19,68	10,235,19	8,68	95,38	15,83	15,39	91,63	0,10	505,90	0,44	0,03	12,74	36,27	1,75	0,70	463,08	68,85	15,36
Harbor 8	8	8,20	13,195,49	6,64	1329,54	0,26	36,73	31,730,47	14,27	94,07	22,16	48,17	155,84	0,36	116,05	3,59	0,32	15,53	57,53	1,64	1,20	710,31	73,48	35,35
Harbor 9	10	8,12	8572,00	0,99	1464,33	0,10	21,48	11,831,00	14,36	37,71	18,66	22,18	76,40	0,12	10,72	0,39	0,05	0,00	26,62	1,80	0,59	507,60	72,13	16,65
Harbor 10	9	8,22	16,656,67	7,88	2850,20	0,23	29,79	19,035,75	10,70	100,70	18,85	33,26	110,40	0,13	168,33	1,25	0,03	19,68	64,40	1,58	2,06	978,63	59,27	34,43

**Table 2:** Correlations between each pair of composition parameters (*n* = 146) (pH: hydrogen potential; Cl: chloride; F: fluoride; SO4: sulphate; Cd: cadmium; Al: aluminum; Cr: chromium; As: arsenic; Cu: copper; Ni: nickel; Pb: lead; Zn: zinc; Hg: mercury; TBT: tributyltin; HAP: polycyclic aromatic hydrocarbons; PCB: polychlorinated biphenyls; Sand: sand content; Mud_2mm: silt content on fraction < 63 µm; Density: bulk density; Total N : total nitrogen; Total P: phosphorus total; DM: dry matter content; OM: organic matter content).

## 4 Experimental Design, Materials and Methods

### 4.1 Sample collection and preparation

Sampling has been established to ensure that the collected sediment samples accurately depict the actual characteristics of the dredged sediment and provide informative value. Therefore, each sample was a composite of at least 5 individual samples taken from 3 distinct zones at 3 different depths in the bottom sediments. They were collected by dredging the 50–80 first centimeters of the harbor seabed with a mechanical shovel. The unweathered sediment was immediately put in 50 L opaque containers under a 10 cm layer of sea water to preserve the anoxic conditions. The composite samples were transported to the laboratory in the 24 h following collection, and then stored in a refrigerated room maintained at 4 °C from time of sampling until geochemical analysis and measurement. The conditions adhered to for the conservation of the sediments make it possible to avoid any reactivity of the sediments and to preserve their chemical properties [3].

### 4.2 Geochemical analyses

Particles size distribution and bulk density were determined according to the NF X 31–107 and the NF ISO 11,464 standards respectively [4,5]. The pH measurements were carried out on pore water after extraction with deionized water, using a solid-to-liquid ratio of 1:2 in accordance with standard EN12457–2 [6]. Chloride, fluoride and sulphate contents were analyzed in leachate subsamples obtained by homogenizing and fragmenting samples into particles smaller than 2 mm according to the EN12457–2 standard [7]. Trace metals in digested sediment samples were determined using an atomic absorption spectrophotometer according to the NF EN ISO 11 885 standard [8]. The technique described in NF EN ISO 1483 was used for Hg analysis [9]. PAH content is represented by the sum of the 16 most common congeners (naphthalene, acenaphthylene, acenaphthene, fluorene, phenanthrene, anthracene, fluoranthene, pyrene, benzo [a] anthracene, chrysene, benzo [b] fluoranthene, benzo [k] fluoranthene, benzo [a] pyrene, dibenzo [a,h] anthracene, benzo [g,h,i] perylene, indeno [1,2,3-c,d] pyrene), analyzed by gas chromatography/mass spectrometry after hexane/acetone extraction according to XPX 33-012 standard [10]. The PCB content is represented by the sum of 7 regulatory congeners (PCB 28, 52, 101, 118, 138, 153 and 180) using the same method as for PAHS [10]. The TBT content was also analyzed by low-resolution mass spectrometry according to the NF EN ISO 23161 standard [11]. Dry matter content was measured according to the NF EN 13,040 standard by drying a subsample at a temperature of 103 ± 2 °C [12]. Organic matter content was determined by calcination at 450 ± 25 °C (standard NF EN 13039) [13]. Total N were analyzed in raw subsamples to avoid gaseous N losses during drying; subsamples were obtained by homogenizing and fragmenting samples into particles smaller than 2 mm. Total N was measured by the Kjeldahl method according to NF EN 13654-1 [14]. Total N analyses were performed in duplicate. Contents of total phosphorus were determined in a subsample dried at 75 ± 5 °C, according to sample preparation standard NF EN 13040 [15]. The analyses were performed using inductively coupled plasma after extraction with aqua regia, according to NF EN 13650 [16].

### 4.3 Data analyses and visualization

Factor analysis represented by a principal component analysis (PCA) was used to identify how compositional parameters (Table 3) covary in order to discriminate the different harbors according to the sediment composition.

**Table 3:** List of variables used for PCA according the content of the dataset (w.w.: wet weight).

File name	Variable name	Content
Geochemical_analysis.csv	Id_sample	Sample identification number
	LAT_RGF93	Latitude of the sampling point
	LONG_RGF93	Longitude of the sampling point
	Sediment_type	harbor type
	pH	hydrogen potential measurements
	Cl	Chloride content (mg Cl kg^−1^ w.w.)
	F	Fluoride content (mg F kg^−1^ w.w.)
	SO4	Sulphate content (mg SO4 kg^−1^ w.w.)
	Cd	Cadmium content (mg Cd kg^−1^ w.w.)
	Cr	Chromium content (mg Cr kg^−1^ w.w.)
	Al	Aluminium content (mg Al kg^−1^ w.w.)
	As	Arsenic content (mg As kg^−1^ w.w.)
	Cu	Copper content (mg Cu kg^−1^ w.w.)
	Ni	Nickel content (mg Ni kg^−1^ w.w.)
	Pb	Lead content (mg Pb kg^−1^ w.w.)
	Zn	Zinc content (mg Zn kg^−1^ w.w.)
	Hg	Mercury content (mg Hg kg^−1^ w.w.)
	TBT	Tributyltin content (µg TBT kg^−1^ w.w.)
	HAP	Polycyclic aromatic hydrocarbons content (mg HAP kg^−1^ w.w.)
	PCB	Polychlorinated biphenyls content (mg PCB kg^−1^ w.w.)
	Sand	Gravel content (> 2000 µm) (%)
	Mud_2mm	Vase (fine fraction <63 µm, over <2 mm) content (%)
	Density	Bulk density (g cm^−3^)
	Total_N	Total nitrogen content (g N kg ^−1^ w.w.)
	Total_P	Total Phosphorus content (mg P2O5 kg^−1^ w.w.)
	DM	Dry matter content (%)
	OM	Organic Matter content (g kg ^−1^ w.w.)

Factor analysis is a set of descriptive statistical methods that allow the maximum amount of information contained in a data table to be presented graphically [17]. This statistical method is adapted to the treatment of mixed data which are relative to a set of individuals described by several groups of variables [18]. PCA is applied to tables that cross-reference rows, representing individuals, with columns representing variables [18]. In this study, the individuals are the sediment samples from the different harbors and the variables are the physicochemical properties, nutrients or chemical contaminants. This was done using the R software R, version: 4.3.2 [17].

## Limitations

Not applicable.

## Ethics Statement

The authors have read and follow the ethical requirements for publication in Data in Brief and confirm that the current work does not involve human subjects, animal experiments, or any data collected from social media platforms.

## CRediT authorship contribution statement

**Garry Dorleon:** Formal analysis, Conceptualization, Methodology, Writing – original draft, Writing – review & editing, Visualization, Data curation. **Isabelle Techer:** Conceptualization, Methodology, Validation, Investigation, Data curation, Writing – original draft, Visualization, Supervision, Project administration. **Sylvain Rigaud:** Conceptualization, Methodology, Validation, Writing – original draft, Visualization, Supervision, Data curation, Project administration.

## Data Availability

Geochemical characterization data of harbors dredged sediments in the Occitanie region (southern France) (Original data) (Recherche Data Gouv).
